# Current Interventions for People Living with HIV Who Use Alcohol: Why Gender Matters

**DOI:** 10.1007/s11904-021-00558-x

**Published:** 2021-06-10

**Authors:** Wendee M. Wechsberg, Felicia A. Browne, Courtney Peasant Bonner, Yukiko Washio, Brittni N. Howard, Isa van der Drift

**Affiliations:** 1 Substance Use, Gender, and Applied Research Program, RTI International, Research Triangle Park, NC USA; 2 Gillings School of Global Public Health, University of North Carolina, Chapel Hill, NC USA; 3 Department of Psychology, North Carolina State University, Raleigh, NC USA; 4 Department of Psychiatry and Behavioral Sciences, Duke University School of Medicine, Durham, NC USA

**Keywords:** Alcohol misuse, ART adherence, Gender, Viral suppression, Women living with HIV

## Abstract

**Purpose of Review:**

Alcohol is the most misused substance in the world. For people living with HIV (PLWH), alcohol misuse may impact ART adherence and viral suppression. This review of the most recently published alcohol intervention studies with PLWH examines how these studies considered gender in the samples, design, and analyses.

**Recent Findings:**

Three searches were conducted initially, and 13 intervention studies fit our criteria with alcohol outcomes. In general, most studies did not consider gender and had used small samples, and few demonstrated significant efficacy/effectiveness outcomes. Five studies considered gender in their samples or analyses and/or were woman-focused with larger samples and demonstrated significant outcomes.

**Summary:**

It is essential for women who misuse alcohol to not only be well represented in alcohol and HIV research but also for studies to consider the barriers to reaching them and their contextual demands and/or co-occurring issues that may affect participation and outcomes in intervention research.

**Supplementary Information:**

The online version contains supplementary material available at 10.1007/s11904-021-00558-x.

## Introduction

Although the world had strived for the 90-90-90 treatment target by the end of 2020, we are not yet there, and the global push for the 95-95-95 treatment target may be temporarily out of reach given that the COVID-19 pandemic has impeded the world’s economies [1] and HIV treatment and prevention [2–4]. Additionally, alcohol, the most widely misused substance in the world [5], may be a key barrier to reaching this goal [6, 7], especially when coupled with challenges related to the COVID-19 pandemic [2–4].

Specifically, alcohol misuse may impact engagement and retention of people living with HIV (PLWH) in the HIV treatment cascade, which is critical to ending the HIV epidemic [8, 9]. Estimates suggest that 35 to 40% of PLWH may misuse alcohol [10]. Furthermore, myriad challenges often intersect with alcohol misuse that affects the health and well-being of PLWH [10]. If untreated, alcohol use disorder (AUD) can contribute to reduced adherence to antiretroviral therapy (ART), retention in HIV care [11, 12], and virologic response to ART, increasing the risk of forward transmission. AUD also ultimately increases mortality among PLWH [13]. Daily alcohol misuse is associated with reduced survival of 6 years among PLWH [13]. Consequently, the intersecting epidemics of alcohol misuse and HIV should be addressed together to help PLWH reach viral suppression and reduce transmission [14]. However, it may not be as simple as this.

Research is growing to address the intersection of alcohol misuse among PLWH [15]. The field has rapidly advanced and developed behavioral [15–17], pharmacological [18, 19], and biobehavioral interventions [20, 21] to reduce alcohol misuse among PLWH, with the National Institute on Alcohol Abuse and Alcoholism (NIAAA) leading this effort [14]. Intervention approaches also have sought to reach key populations, including sexual minorities [22–24]. Intervention research, although nascent, also has extended its focus to cisgender women [25–27] and transgender women [25] living with HIV to address alcohol misuse.

However, this was not always the case, and the contemporary definition of gender is not binary (male-female), although most studies write in this fashion. For many years, women were excluded from or minimally involved in research [28]. For example, as recently as the 1980s, women were excluded from trials of HIV drugs [28]. Not until 1993 was the inclusion of women in National Institutes of Health research written into law [28, 29]. Importantly, alcohol misuse among women has recently gained greater recognition [30–33]. Including women in HIV and alcohol intervention research is critical because women’s physiological response to HIV and alcohol is different than men’s response; consequently, intervention effects may differ [34, 35]. NIAAA has highlighted the biological differences experienced by women when they drink and is in the process of reevaluating the recommended alcohol threshold for women [36].

This exclusionary trend also applies to pregnant populations, one of the most vulnerable populations to HIV, that historically have been excluded from alcohol research or addressed in research as a special population [37–39]. The need for more HIV and alcohol misuse research with this population is evident given the implication of prenatal drinking in fetal alcohol spectrum disorders (FASD) and other associated syndemic issues related to gender-based violence and ART adherence [40–47]. NIAAA has been supporting FASD research for almost 50 years [48] among First Americans and in South Africa where heavy drinking is prominent [49–52]. Nonetheless, more research is needed.

The impact of alcohol misuse and HIV has greater adverse consequences for women compared with men [6, 34]. Hazardous alcohol use among women living with HIV (WLWH) is associated with HIV risk behaviors [53] and adverse health and HIV treatment outcomes [54], especially in economically underserved communities where heavy drinking may persist [54, 55]. Moderate drinking has been found to be associated with increased vaginal shredding among WLWH on ART [56]. Increased alcohol intake has been found to be associated with lower ART adherence [7]. Given the associations among alcohol misuse, vaginal shedding, and ART adherence for WLWH, moderate to severe alcohol use may increase risk of forward HIV transmission [56–59]. These findings illustrate the need for intervention trials to not only include women but also include them in sufficient numbers and to incorporate a gender lens for intervention approaches and analyses.

Given the importance of understanding what has worked in the most recent intervention research literature with PLWH who misuse alcohol, this review also highlights intervention studies focused on alcohol outcomes. However, essential to this review was our search with a gender lens in seeking sample proportions and analyses. Consequently, the core of this review is a search for gender interventions and why gender matters. Understanding the multidimensional issues that WLWH face and alcohol misuse is complex.

## Methods

To ensure our review reached saturation of the available literature, multiple literature searches were conducted between December 2020 and February 2021 utilizing PubMed, Embase, and Web of Science scientific databases, as well as confirmatory checks in the NIH RePORTER and ClinicalTrials.gov. We included publications that met the following criteria: (1) published between 2018 and early February 2021; (2) reported or proposed alcohol outcomes for a randomized trial; (3) equal to or greater than 50% of the sample comprised PLWH; (4) the sample included cisgender or transgender women; and (5) written in English. We then reviewed the publications to determine whether there was an adequate sample size for a rigorous intervention trial [59, 60] and gender was accounted for in the study design (including intervention) and/or analysis (e.g., stratified analyses, gender as a moderator). Protocols were only included in this review if the proposed procedures considered women in the approach, intervention, design or outcomes, or if the intervention was developed for and about women and addressed alcohol as one of the proposed outcomes.

We conducted the search for publications in three phases. The initial search was conducted with a specific focus on seeking publications evaluating alcohol interventions for WLWH, including pregnant women; the second search expanded the criteria to alcohol interventions with PLWH; and the third search expanded the criteria to include a wide range of alcohol, drinking, and HIV-related terms to ensure that all appropriate publications were identified (see Appendix 1 for the entire search term list and criteria for each search).

After identifying published articles based on the search terms, research staff downloaded the results into an EndNote database and used a priori criteria to narrow the search using a categorical folder system. Although we made the search as comprehensive as possible, we did not search conference abstracts, theses, or dissertations; nor did we contact authors of unpublished articles. Our team reviewed the abstracts generated by the search. If the abstract indicated that the article might meet the specified criteria, it was reviewed in its entirety to ensure the inclusion criteria were met. Promising articles were then separated and presented to all of the present authors for their final review and approval for inclusion in this review.

**Fig. 1:**
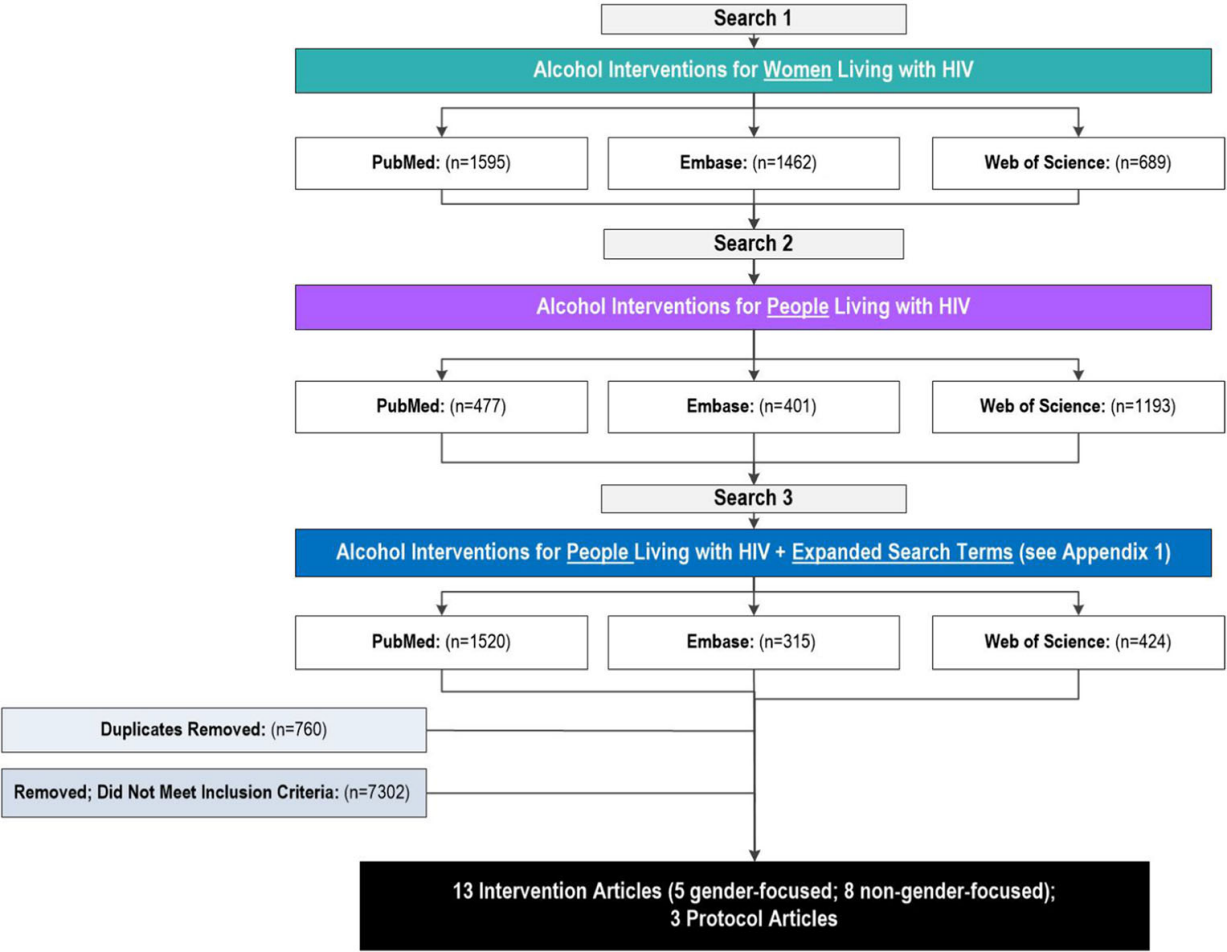
Search diagram

As shown in Fig. 1, the combined searches returned 8076 results. Of these, 7302 were excluded because they did not meet the eligibility criteria. A majority of the articles excluded during the abstract review did not focus on PLWH, were HIV prevention studies (and thus did not include a large percentage of PLWH), were cross-sectional, reported secondary data analysis from a prior trial, or did not report or propose alcohol outcomes. As a result of these searches, 13 intervention articles (8 non-gender and 5 gender-focused) and 3 protocols were included.

## Results

### Non-gender Studies

Eight of the identified studies evaluated the efficacy or effectiveness of interventions to reduce alcohol use among PLWH but did not use a gender lens in the design or analyses [21, 61–67]. The sample sizes of these studies ranged from 93 to 614, with 75% reporting sample sizes of fewer than 250. Of the 1897 participants across these 8 studies, only 7.9% were female or gender non-conforming, with 62.5% of the studies reporting more than 95% male participants. All the interventions evaluated incorporated motivational interviewing. Two interventions included cognitive behavioral therapy (CBT) components [63, 67] and 3 interventions included medication-assisted treatment for alcohol use [21, 61, 62]. All interventions were delivered individually, with one intervention reporting additional optional group intervention components [63].

All studies used self-report alcohol assessments, such as the Alcohol Use Disorders Identification Test (AUDIT) or the Alcohol Smoking and Substance Involvement Screening Test (ASSIST). Six studies used the Timeline Followback (TLFB) to assess outcomes such as days of drinking, drinks per drinking day, and number of heavy drinking days in the past month [21, 61–64, 66]. Other self-report measures to assess alcohol-related outcomes included AUDIT and ASSIST. Also, 3 of the non-gender studies used phosphatidylethanol (PEth) biomarkers to assess alcohol abstinence [21, 61, 62]. Some studies included in this review also measured viral load using HIV RNA and the cut-off for viral suppression ranged from <20 to <50 copies/ml. Although most studies reported that participants reported reduced alcohol use at follow-up, only 3 studies demonstrated statistically significant intervention effects [63, 64, 67]. Go and colleagues conducted a trial comparing a culturally adapted motivational enhancement therapy and a brief alcohol intervention to standard of care among 440 PLWH in Thai Nguyen, Vietnam [63]. The findings from this study indicated that both intervention arms reported significantly greater abstinence and reductions in alcohol use and greater viral suppression at follow-up compared with participants in the standard of care arm. Madhombiro and colleagues demonstrated the efficacy of a combined motivational interviewing and CBT intervention to reduce AUDIT scores among 234 PLWH in Zimbabwe [67]. Lastly, Naar and colleagues reported the effectiveness of Health Choices, a motivational interviewing-based intervention, among 183 youth living with HIV that was implemented in and tested intervention delivery (home as compared with clinic settings) [64]. This study demonstrated that youth who received the intervention in a clinic setting were significantly more likely to maintain reductions in self-reported alcohol use and viral suppression (Table 1).

**Table 1:** Interventions with people living with HIV who use alcohol (non-gender element)

Citation	Population/country	Study arms	Alcohol use outcome measures	Viral load outcome measures	Findings
Edelman et al., 2019a [21]	***N*=128** (97.5% male, 78.9% Black) Veterans living with HIV who met DSM-IV criteria for AUD; multisite trial; *USA*	**Intervention:** ISAT Interventions: Brief Negotiated Interview; 4 individual sessions of psychologist-delivered Motivational Enhancement Therapy + Medication-Assisted Treatment; Stepped treatment over 24 weeks **Standard of Care:** Annual screening via AUDIT-C; Brief interventions or referral to addiction treatment and health handouts	• Percentage of days abstinent (TLFB) • PEth blood levels (DBS) • Past 30-day abstinence (TLFB) • Drinks per week (TLFB) • Percentage of participants with no heavy drinking days (men: >5 drinks/day; women: >4 drinks/day; TLFB) • Mean number of drinks per drinking day (TLFB)	• Undetectable plasma HIV viral load (HIV RNA <50 copies/ml)	• At 24- and 52-week follow-up: Both study arms reported increased alcohol abstinence; No significant differences in alcohol use or viral load outcomes between study arms
Edelman et al., 2019b [62]	***N*=95** (99% male; 85% Black) Veterans living with HIV and liver disease who consumed alcohol in the past 30 days; multisite trial; *USA*				• At 24- and 52-week follow-up: Both study arms reported increased alcohol abstinence; No significant differences in alcohol use or viral load outcomes between study arms
Edelman et al., 2020 [61]	***N*=93** (97% male; 78.7% Black) Veterans living with HIV at risk for alcohol use; multisite trial; *USA*				• At 24- and 52-week follow-up: Both study arms reported increased alcohol abstinence; No significant differences in alcohol use or viral load outcomes between study arms
Go et al., 2020 [63]	***N*=440** (96.8% male) People living with HIV receiving ART and with hazardous alcohol use; *Vietnam*	**Intervention:** Combined intervention of culturally adapted Motivational Enhancement + Cognitive Behavioral Therapy; 6 individual face-to-face sessions and 3 optional group sessions **Intervention:** Culturally adapted Brief Alcohol Intervention; 2 face-to-face sessions and 2 booster telephone sessions **Standard of Care:** Recommendation to reduce alcohol use; referral to harm reduction services and treatment for HBV and HCV, STIs, and TB	• Self-reported percentage of days abstinent (TLFB) • Number of drinks per day (TLFB) • Heavy drinking days (defined as >4 drinks for men and >3 drinks for women; TLFB)	• Viral suppression (HIV RNA <20 copies/ml)	• At 3-, 6-, and 12-month follow-up, participants in **both intervention arms reported a significantly larger increase in days of abstinence and a significantly lower number of heavy drinking days compared with participants in the SOC arm** • Participants in the Brief Alcohol Intervention arm were more likely to be virally suppressed than participants in the SOC arm (89.2% vs. 78.1%) at 12-month follow-up • At 3- and 6-month follow-up, participants in **both intervention arms reported significantly greater reductions in drinks per drinking day compared with participants in the SOC arm, and at 12-month follow-up, participants in the combined intervention arm reported significantly greater reductions in drinks per drinking day compared with participants in the SOC arm**
Madhombiro et al., 2020 [67]	***N*=234** (78.6% male) People living with HIV on ART who have an AUD; *Zimbabwe*	**Intervention:** MICBT; 8 to 10 individual sessions, lasting 45–60 min each **Enhanced usual care:** Alcohol-use module from the WHO Mental Health Gap intervention guide	• AUDIT score	• Adherence (percentage of scheduled visits for medication refills in the past 3 months) • Viral suppression (<40 copies/ml) • CD4 count	• **Intervention participants had significantly greater reductions in AUDIT scores at 6-month follow-up compared with participants in the control group** • Both study groups had a significant decrease in viral load, but no statistically significant difference between the study arms at 6-month follow-up • No statistically significant reduction in CD4 count within groups and/or between study arms at 6-month follow-up
Stein et al., 2020 [66]	***N*=110** (81.8% male) People living with HIV or current or previous HCV infection and reported consuming at least 4 alcoholic drinks per week in the past month; *USA*	**Intervention:** REACH with motivational enhancement elements; 7 individual phone sessions, 20-30 min each, every 3 months for 18 months **Intervention:** Brief advice with 7-sessions about the HIV-HCV-related risks of alcohol use	• Drinking days (TLFB) • Number of drinks per day (TLFB) • Addiction Severity Index	• HIV RNA viral load	• Drinks per drinking day significantly reduced in both study groups, but reductions were not statistically significant between study groups • No statistically significant treatment effects for any alcohol use outcome • No HIV outcomes reported
Satre et al., 2019 [65]	***N*=614** (96.9% male) People living with HIV who self-reported unhealthy alcohol use in the past year; *USA*	**Intervention:** Motivational Interviewing; one 45-min in-person individual session at the clinic followed by two 20-min telephone sessions **Intervention:** Email Feedback; one email sent via a secure patient portal messaging function **Standard of Care**: Routine HIV primary care; screening for unhealthy drinking based on NIH-recommended thresholds; referrals to specialty addiction services	• Number of drinking days in the past 30 days • Addiction Severity Index • Alcohol Importance Ruler, and Confidence Ruler	• Self-reported ART adherence • HIV RNA levels	• All arms demonstrated a statistically significant decrease in alcohol use outcomes, but no significant difference between arms • Among participants who reported that reducing drinking was of low importance (*n*=334), participants in the motivational interviewing arm were **significantly less likely to have any heavy drinking days than participants in the SOC arm** at 12-month follow-up
Naar et al., 2020 [64]	***N*= 183** (79.2% male, 13.7% female, 7.1% transgender or gender nonconforming) adolescents (aged 16 to 24 years) living with HIV; 5 sites in *USA*	**Intervention:** Adapted Healthy Choices Intervention – Home: 4 30-min individual sessions over 10 weeks delivered at home or in community by a paraprofessional **Intervention:** Adapted Healthy Choices Intervention – Clinic: 4 30-min individual sessions over 10 weeks delivered in home by a paraprofessional	• Severity of problems (ASSIST) • Number of drinks per week (TLFB)	• Undetectable plasma HIV viral load (HIV RNA <20)	• Participants in the clinic delivery group maintained reductions in alcohol • The clinic delivery group had **significantly greater reductions in viral load over each post-intervention follow-up**

Note: *ART*, antiretroviral therapy; *ASSIST*, Alcohol Smoking and Substance Involvement Screening Test; *AUD*, alcohol use disorder; *AUDIT-C*, Alcohol Use Disorders Identification Test-Consumption; *DBS*, dried blood spot; *HBV*, hepatitis B virus; *HCV*, hepatitis C virus; *ISAT*, Integrated Stepped Alcohol Treatment; *NIH*, National Institutes of Health; *MICBT*, Motivational Interviewing + brief Cognitive Behavioral Therapy; *PEth*, phosphatidylethanol; *RNA*, ribonucleic acid; *SOC*, standard of care; *STI*, sexually transmitted infection; *TB*, tuberculosis; *TLFB*, Timeline Followback; *WHO*, World Health Organization

Bold font in the Findings column indicate statistically significant differences between arms

### Gender Studies

Five of the identified studies evaluated the efficacy or effectiveness of interventions to reduce alcohol use among PLWH and used a gender lens in the design and/or analyses [26, 27, 68–70]. The sample sizes in this group of studies ranged from 194 to 641. Three of the 5 studies comprised all women, with 1 study sample reporting 46% women and 1 study sample reporting 51.5% women [26, 27, 69]. A variety of intervention approaches were represented, including pharmacotherapy for AUD, the information-motivation-behavior-skills framework, CBT, and feminist-based social cognitive theory.

All studies used self-report measures to assess alcohol use outcomes. The TLFB was the assessment used most. PEth was only used in 1 study as a measure of alcohol use. Additionally, all studies assessed some proportion of viral load among participants, with viral suppression cut-offs at <200 copies/ml in 2 studies and 1360 copies/ml in 1 study. All studies reported significant intervention effects for alcohol use outcomes. Cook and colleagues conducted a trial testing the efficacy and safety of oral naltrexone among 194 primarily African American WLWH and found that participants in the naltrexone intervention arm were significantly more likely to report lower levels of unhealthy drinking at short-term follow-up but not at the follow-up endpoint compared with participants in the placebo arm. HIV viral suppression was also significantly better for women who reduced drinking as compared with women who continued unhealthy alcohol use at 4 months [26]. Huis and colleagues investigated the effects of an information-motivation-behavior-skills intervention with personalized feedback and a brief counseling session compared with a health education leaflet on alcohol use among 560 PLWH in South Africa [68]. This study found that participants in the intervention and counseling arms had significantly greater reductions in AUDIT scores from baseline to follow-up compared with participants in the control arm. Additionally, when comparing men and women, men had a greater percentage reduction in AUDIT scores compared with women. Papas and colleagues tested the efficacy of a cognitive-behavioral therapy (CBT) intervention to reduce alcohol use among 614 outpatients living with HIV with hazardous drinking enrolled at an HIV clinic in Eldoret, Kenya. They found significantly lower percentages of drinking days and drinks per drinking day in the CBT arm than the healthy life-styles educational intervention (HL) arm overall and at all study phases. Furthermore, adherence and competence scores in both the CBT and HL conditions did not differ significantly by gender [70].

Wechsberg and colleagues conducted a community-based cluster randomized trial evaluating the efficacy of a woman-focused HIV and substance use intervention to reduce substance use, including alcohol, and other risk behavior among 641 women who engaged in substance use [27]. Of the 641 women in the study, 317 were living with HIV and reported engaging in alcohol use. The overall trial demonstrated efficacy of the intervention to reduce heavy drinking and the number of days that participants drank in the past month. Chi-square tests of independence using dried blood spot samples indicated there was no difference in the proportion of participants who had nondetectable viral loads at 6-month follow-up (*n*=118; *p*=0.83). However, there was a significant difference at 12-month follow-up (*n*=172; *p*=0.01). Approximately 53% of participants in the Women’s Health CoOp Plus arm (*n*=50) had nondetectable viral loads compared with 47% of participants in the HIV counseling and testing arm (*n*=45) at 12-month follow-up. The results from a sensitivity analyses among 317 participants who were living with HIV and reported engaging in alcohol use revealed that the intervention also was efficacious among this subsample and significantly reduced the number of drinks per drinking day and number of days of alcohol use in the past month.

Lastly, Wechsberg and colleagues [69] conducted an implementation science hybrid modified stepped-wedge design implementing the Women’s Health CoOp (WHC) intervention in matched health departments and substance abuse treatment clinics with 480 WLWH over four cycles in Cape Town, South Africa. Compared with cycle 1, women in cycle 4 were significantly less likely to report AUD risk at 6-month follow-up. Additionally, compared with women in cycle 1, women in cycle 4 were significantly more likely to report taking ART at follow-up with over 90% adherence at 6-month follow-up. The likelihood of taking ART increased as women were enrolled in the later cycles and the risk of AUD decreased. The WHC intervention increased ART adherence and reduced alcohol use overall (Table 2).

**Table 2:** Interventions with people living with HIV who use alcohol (gender element)

Citation	Population/setting	Study arms	Alcohol use outcome measures	Viral load outcome measures	Findings
Cook et al., 2019 [26]	***N*=194** Women living with HIV who reported consuming >7 drinks/week or >3 drinks on 1 day at least twice; *USA*	**Intervention:** Naltrexone pill taken orally once a day; 4 months **Placebo**	• The average number of drinks per week (TLFB) • Number of days of abstinence (TLFB) • Number of binge-drinking days in the past 30 days (TLFB) • PEth blood levels (DBS)	• Self-reported ART adherence • CD4+ cell count • Undetectable plasma HIV viral load (HIV RNA <200 copies/ml)	• **Intervention participants had significantly lower levels of unhealthy drinking at 1-month and 3-month follow-up**, but not at 4-month follow-up • No significant difference in odds of reducing or quitting drinking or other alcohol outcomes between study arms at follow-points • Adherence and viral load outcomes did not differ by group
Huis in’t Veld et al., 2019 [68]	***N*=560** (53.9% male, 46.1% female) Adults with HIV-1 and who visited the 3 selected HIV clinics; *South Africa*	**Intervention:** Information-Motivation-Behavioral Skills Model-based intervention**;** Health education leaflet on responsible drinking Personalized feedback on AUDIT scores **Brief counseling session** on reducing excessive drinking; individual; 1 session **Control: Health education leaflet** on responsible drinking	• 10-item AUDIT	• CD4+ count; viral load • ART adherence	• Intervention and counseling session participants have a significant reduction in AUDIT scores; no significant difference between study groups • Intervention participants’ mean last measured CD4 count was significantly lower at time point 1 but not at time point 2 • Intervention did not influence other HIV outcomes
Papas et al., 2021 [70]	***N*=614** (48.5% male) Adults enrolled as an AMPATH HIV outpatient with hazardous drinking; *Kenya*	**Intervention**: CBT intervention consisting of 6 weekly 90-min group sessions **Healthy Life-styles (HL)**: educational intervention encouraging healthy lifestyle choices consisting of 6 weekly 90-min group sessions	• Percentage of drinking days (TLFB) • Drinks per drinking day (TLFB) • Asking participants how much money they spent on personal consumption	• Self-reported ARV adherence • HIV RNA concentration (<40 copies/ml)	• **Significantly lower percentage of drinking days and drinks per drinking day in CBT than HL overall and at all study phases** • Adherence and competence scores in both CBT and HL conditions did not differ significantly by gender
Wechsberg et al., 2019 [27]	***N*=641** Black African women (aged 15 or older) in 14 communities who reported weekly use of at least one substance, which could be alcohol; subanalyses conducted with *N*=317 women living with HIV who reported using alcohol; *South Africa*	**Intervention:** Women’s Health CoOp Plus (WHC+) and **s**tandard HIV counseling and testing; 2-h-long individual sessions with personalized action plan **Standard HIV Counseling and Testing**	• Average number of drinks per day	• Undetectable viral load (<1360 copies/ml) for subsample	• Among the subsample with viral load data, the WHC+ arm was statistically significantly more likely to have a nondetectable viral load (*p*=0.01) at 12-month follow-up; this difference was not statistically significant at 6-month follow-up • **Women in the WHC+ arm reported significantly fewer drinks per day and days of drinking in the past month than participants in the comparison condition**
Wechsberg et al., 2021 [69]	***N*=480** Women living with HIV (aged 18 to 45) who report the use of at least one drug at least weekly in the past 3 months (one of which could be alcohol); *South Africa*	**Intervention:** Women’s Health CoOp (WHC): 2-session empowerment-based group workshop addressing HIV, STIs, TB, condoms, sexual risk reduction, alcohol and other drugs, violence, and negotiation skills WHC implemented in health and substance use clinics over 4-cycles via a modified stepped- wedge design implementation science trial	• Alcohol use (self-reported frequency, amount, binge drinking to calculate heavy alcohol use [4 or more drinks on any given day, and 7 or more drinks per week])	• ART initiation and adherence • Awareness of CD4 count	• Compared with cycle 1, women in cycle 4 were **significantly less likely to AUD risk at 6-month follow-up** • Compared with women in cycle 1, women in cycle 4 were **significantly more likely to report taking ART in the past 6 months at follow-up** • Likelihood of taking ART increases as women are enrolled in the later cycles and the risk of AUD decreases • WHC increased ART adherence and reduced alcohol use

Note: *ART*, antiretroviral therapy; *AUD*, alcohol use disorder; *AUDIT*, Alcohol Use Disorders Identification Test; *CBT*, cognitive behavioral therapy; *DBS*, dried blood spot; *PEth*, phosphatidylethanol; *STI*, sexually transmitted infection; *TB*, tuberculosis; *TLFB*, Timeline Followback

Bold font in the Findings column indicate statistically significant differences between arms

### Protocols for Ongoing Gender-Focused Studies

We also identified 3 protocols of ongoing studies currently considering gender in their evaluation of interventions that seek to reduce alcohol use among PLWH [71–73]. The 3 protocol papers reviewed include motivational interviewing, computer-based interventions, and the woman-focused intervention approaches. The planned sample sizes for these studies range from 60 to 200.

Two studies did not explicitly state a sample size goal for the recruitment of women, and participants are eligible regardless of gender. One study will recruit women exclusively. Two studies will use self-report alcohol measures (ASSIST or AUDIT) and 1 of these studies also will use urinalysis to assess alcohol outcomes. One study will exclusively use PEth and ethyl glucuronide (EtG) biomarker testing to assess alcohol outcomes. Two studies also will assess HIV outcomes using viral load, CD4+ cell count, and ART adherence measures (Table 3).

**Table 3:** Protocols

Citation	Planned population/setting	Study arms	Proposed alcohol outcomes	Proposed HIV and viral load outcomes	Proposed findings gender-focused
DiClemente et al., 2021 [71]	***N*=200** Adult women living with confirmed HIV/HCV coinfection aged 18 to 45 who currently use alcohol and who are selected from HIV care clinics; *Russia*	**Intervention: Standard of Care + Brief computerized intervention** addressing relevant health topics concluding with individual visits with study clinicians to set alcohol consumption goals **Standard of Care:** Women are routinely asked about their alcohol consumption and referred to treatment facilities if necessary; health educational brochure relevant to HIV/HCV and alcohol	• PEth levels (8 ng/ml) • EtG levels (500 ng/ml)	• HIV viral load (copies/ml) • CD4+ cell count	• To determine if a computer-based intervention that could provide better patient confidentiality and increased accessibility is efficacious in improving alcohol and HIV/HCV outcomes
Kane et al., 2020 [72]	***N*=180** People living with HIV with high-risk alcohol use and potential mental health or other substance use comorbidities from two hospitals with large HIV clinics; *Zambia*	**Intervention:** CETA adapted for HIV clinical settings; CBT for substance use reduction; IND; 6 to 12 1-h sessions **Intervention:** Alcohol Behavioral Intervention adapted from CETA elements for Substance Use Reduction	• 10-item AUDIT • ASSIST substance use measurement	• No proposed adherence or viral load outcomes	• Will reveal the effectiveness of an intervention addressing substance use and other comorbidities in an HIV clinic setting in sub-Saharan Africa
Magidson et al., 2020 [73]	***N*=60** Adults living with HIV who are currently on ART but struggling with adherence and with moderate substance use from an HIV clinic in Khayelitsha; *South Africa*	Hybrid Type 1 effectiveness implementation trial **Intervention: LifeSteps + Motivational Interviewing;** Relapse prevention skills; 6 sessions lasting between 45 min and 1 h **Standard of Care:** Referral to substance use treatment services	• Urinalysis and self-report • WHO ASSIST	• ART adherence measured through Wisepill • Viral load (copies/ml)	• Determine whether this evidence-based intervention can be integrated into HIV care settings, considering workforce shortage in South Africa • Establish whether this intervention successfully addresses both HIV treatment and substance use

Note: *ASSIST*, Alcohol Smoking and Substance Involvement Screening Test; *AUDIT*, Alcohol Use Disorders Identification Test; *CBT*, cognitive behavioral therapy; *CETA*, common elements treatment approach; *EtG*, ethyl glucuronide; *HCV*, hepatitis C virus; *PEth*, phosphatidylethanol; *WHO*, World Health Organization

## Discussion

So why is gender important in research? Women are the face of HIV globally because of gender norms and inequities [74]. Women face multiple contextual and structural barriers that may interfere with their ability to seek treatment for alcohol misuse and HIV care [55, 75, 76], let alone participate in research. Additionally, comprehensive gender-focused approaches are rare [77] and far too often childcare needs disproportionately hinder access to healthcare while also facing financial strain in less equitable employment as compared with men [76, 78].

Additionally, women who misuse alcohol may experience multiple challenges, including stigma [75]. Staff may have negative attitudes and lack person-centered care toward women who use alcohol and who are seeking treatment [75]. More recently, the ability to validate ART adherence and demonstrate reductions in viral load transmission has become a major component of intervention outcomes, and therefore the need to address alcohol as an interfering factor to ART adherence is critical [79–81]. However, the cost for conducting large trials may be a constraint.

For decades, studies focused on women who were pregnant and used alcohol and consequent fetal alcohol effects. In fact, these women have not been treated as a whole person, for example, by taking into account the fact that they often face violence from partners, especially during pregnancy [43, 82]. However, a recent study reviewing HIV medication adherence among women who are pregnant and living with HIV in sub-Saharan Africa brought attention to the importance of supporting not only the woman but also her partner and the family in addressing fear, stigma, and discrimination around HIV [39], as ART initiation and adherence can be compromised by lack of support from partners and friends and their alcohol use [42].

A majority of the interventions were conducted domestically within the USA; however, interventions conducted internationally were culturally adapted to suit the environment and population [63, 67, 69, 70]. Regardless of location, most of the interventions contained a brief interview component. Studies conducted globally were more likely to identify significant reductions in alcohol consumption. Furthermore, the internationally focused studies were more likely to report higher levels of viral suppression and ART adherence, although few of the studies examined overall reported positive HIV outcomes. It is difficult to attribute these outcomes purely to whether a study was conducted internationally or domestically as many of the international studies had larger sample sizes—as they were conducted in areas with high HIV prevalence—thus they may have been more likely to report robust and significant outcomes. Lastly, the internationally focused interventions were also more likely to have a gender component or be gender-focused as compared with those conducted domestically.

It is a concern why some NIH- and NIAAA-supported studies have had such small participation of women relative to men. As gender intervention researchers, we believe there is much to do in addressing alcohol use, ART adherence, and other associated behavioral and psychological issues, including stigma and discrimination, other drug use, and gender-based violence. To better understand this, we reviewed numerous studies that address these intersectional issues and found that either they were HIV prevention studies, were not rigorous intervention studies, or did not have alcohol as an outcome variable. We acknowledge the importance of how motivational interviewing and a personalized approach can work to meet an individual where they are for engagement, but several of these approaches lacked gender sensitivity. Developing personalized action plans and empowering a woman to take action have been essential elements of the Women’s Health CoOp, a woman-focused intervention for over 25 years for reducing alcohol and other drug misuse and more recently increasing ART adherence [27, 69, 83, 84].

Nonetheless, how women are reached, whether an effort is made to oversample for adequate analyses, and sensitive consideration of their contextual needs and comorbid symptoms are essential to shaping a more just scientific response to the call for “why gender matters” More studies are needed with sexual minorities of women because they have higher rates of alcohol use, violence directed at them, HIV, and suicide [85–87]. Understanding the spectrum of gender is evolving as the world becomes more conscious and accepting.

The landscape of gender-focused interventions and applied research with women who misuse alcohol and other drugs, especially those living with HIV, requires not only sensitivity but also culturally congruent applications with the voice and involvement of the participants. As applied scientists over several decades, we have moved from the behavioral to biobehavioral, with treatment combinations and mixed methods to understand and conduct research that is meaningful and more impactful with many of the participants at the table, including peer advisory and community collaborative boards. However, there remains much to learn about what works best with each population.

## Conclusion

In summary, alcohol misuse continues and FASD still is a public health issue [88–90]. Much can be done globally in intervention research with WLWH who misuse alcohol. Yet, intervention research cannot be everything to all. We also need to listen to women’s stories, consider gendered measures and stratified approaches, empower women be more educated about their HIV status, reduce barriers to ART and alcohol treatment, measure efficacy and effectiveness to know what will work, and identify mediators and moderators that affect change to ultimately understand what can be sustained in the real world [91]. Gender matters and women matter because they are mothers of the next generation.

## Supplementary Information

ESM 1 (DOCX 13 kb).

## Data Availability

The search terms used for this review are available in the supplementary material of this article.

## Abbreviations

ART: antiretroviral retroviral therapy
ASSIST: Alcohol Smoking and Substance Involvement Screening Test
AUD: alcohol use disorder
AUDIT: Alcohol Use Disorders Identification Test
CBT: cognitive behavioral therapy
EtG: ethyl glucuronide
FASD: fetal alcohol spectrum disorders
NIAAA: National Institute on Alcohol Abuse and Alcoholism
PLWH: people living with HIV
TLFB: Timeline Followback
WLWH: women living with HIV
