# Supplementary material for: Current Interventions for People Living with HIV Who Use Alcohol: Why Gender Matters
Source: Curr HIV/AIDS Rep. 2021 Jun 10;18(4):351–64. doi: 10.1007/s11904-021-00558-x (PMC8190977; doi:10.1007/s11904-021-00558-x)
Supplement: Supplementary file 1 — (DOCX 13 kb). [file 11904_2021_558_MOESM1_ESM.docx]

**Appendix 1. Search Terms and Criteria**

**Search 1: Alcohol Interventions for Women Living with HIV**

(((((("HIV"[Title/Abstract] OR "AIDS"[Title/Abstract] OR "HIV/AIDS"[Title/Abstract] OR "human immunodeficiency virus"[Title/Abstract]) AND "women living with"[Title/Abstract] OR "women"[Title/Abstract]) AND ("substance"[Title/Abstract] OR "drink"[Title/Abstract] OR "alcohol"[Title/Abstract])) OR "other drugs"[Title/Abstract]) AND "intervention"[Title/Abstract] AND 2018/01/01:3000/12/31[Date - Publication]))))))

**Search 2: Alcohol Interventions for People Living with HIV**

(((((("HIV"[Title/Abstract] OR "AIDS"[Title/Abstract] OR "HIV/AIDS"[Title/Abstract]) OR "people living with HIV"[Title/Abstract] AND ("alcohol"[Title/Abstract] OR "drinking"[Title/Abstract] AND "intervention"[Title/Abstract]) OR "program"[Title/Abstract]) OR "programme"[Title/Abstract]) OR "trial"[Title/Abstract]) AND 2018/01/01:3000/12/31[Date - Publication])))))

**Search 3: Alcohol Interventions for People Living with HIV + Expanded Search Terms**

((((((“people living with HIV"[Title/Abstract] OR “people living with HIV/AIDS"[Title/Abstract] OR “HIV-positive"[Title/Abstract OR “HIV+"[Title/Abstract] OR “HIV Seropositive"[Title/Abstract] OR “HIV-infected"[Title/Abstract] OR “HIV patients" [Title/Abstract] AND “drinking"[Title/Abstract] OR “binge”[Title/Abstract] OR “drinkers”[Title/Abstract] OR “heavy drinking”[Title/Abstract] OR “heavy drinkers”[Title/Abstract] OR “alcoholic beverages” ”[Title/Abstract] OR “alcohol drinking” [Title/Abstract] OR “alcohol abuse”[Title/Abstract] OR “alcoholic”[Title/Abstract] OR “alcohol”[Title/Abstract] OR “alcohol-related disorders”[Title/Abstract] OR “alcoholism”[Title/Abstract] OR “intoxicated”[Title/Abstract] OR “drunk”[Title/Abstract] OR “liquor”[Title/Abstract] AND “intervention”[Title/Abstract] OR “trial” ”[Title/Abstract] OR “program”[Title/Abstract] OR “programme”[Title/Abstract] AND 2018/01/01:3000/12/31[Date - Publication]))))))))))))
